# Use of Artificial Intelligence Methods for Predicting the Strength of Recycled Aggregate Concrete and the Influence of Raw Ingredients

**DOI:** 10.3390/ma15124194

**Published:** 2022-06-13

**Authors:** Xinchen Pan, Yixuan Xiao, Salman Ali Suhail, Waqas Ahmad, Gunasekaran Murali, Abdelatif Salmi, Abdullah Mohamed

**Affiliations:** 1 Department of Art and Design, Shenyang Ligong University, Shenyang 110159, China; 2 Department of Architecture, Tianjin University, Tianjin 300072, China; 3 Department of Architecture and Urban Planning, Shenyang Jianzhu University, Shenyang 110168, China; xiaoyixuan2021@sina.com; 4 Department of Civil Engineering, University of Lahore (UOL), 1-Km Defence Road, Near Bhuptian Chowk, Lahore 54000, Pakistan; salmanalisuhail@gmail.com; 5 Department of Civil Engineering, COMSATS University Islamabad, Abbottabad 22060, Pakistan; 6 Peter the Great St. Petersburg Polytechnic University, 195251 St. Petersburg, Russia; murali_22984@yahoo.com; 7 Department of Civil Engineering, College of Engineering, Prince Sattam Bin Abdulaziz University, Al-Kharj 16273, Saudi Arabia; a.salmi@psau.edu.sa; 8 Research Centre, Future University in Egypt, New Cairo 11845, Egypt; mohamed.a@fue.edu.eg

**Keywords:** concrete, splitting-tensile strength, cracking, building material, construction material

## Abstract

Cracking is one of the main problems in concrete structures and is affected by various parameters. The step-by-step laboratory method, which includes casting specimens, curing for a certain period, and testing, remains a source of worry in terms of cost and time. Novel machine learning methods for anticipating the behavior of raw materials on the ultimate output of concrete are being introduced to address the difficulties outlined above such as the excessive consumption of time and money. This work estimates the splitting-tensile strength of concrete containing recycled coarse aggregate (RCA) using artificial intelligence methods considering nine input parameters and 154 mixes. One individual machine learning algorithm (support vector machine) and three ensembled machine learning algorithms (AdaBoost, Bagging, and random forest) are considered. Additionally, a post hoc model-agnostic method named SHapley Additive exPlanations (SHAP) was performed to study the influence of raw ingredients on the splitting-tensile strength. The model’s performance was assessed using the coefficient of determination (R^2^), root mean square error (RMSE), and mean absolute error (MAE). Then, the model’s performance was validated using k-fold cross-validation. The random forest model, with an R^2^ of 0.96, outperformed the AdaBoost models. The random forest models with greater R^2^ and lower error (RMSE = 0.49) had superior performance. It was revealed from the SHAP analysis that the cement content had the highest positive influence on the splitting-tensile strength of the recycled aggregate concrete and the primary contact of cement is with water. The feature interaction plot shows that high water content has a negative impact on the recycled aggregate concrete (RAC) splitting-tensile strength, but the increased cement content had a beneficial effect.

## 1. Introduction

The splitting-tensile strength is an important mechanical property of concrete that considerably impacts on the cracking size and extent in concrete structures. As concrete is weak under tension, a pre-assessment of its splitting-tensile strength is necessary to conduct [1,2]. As per the theory of brittle fracture, the specimen failure starts from the largest crack oriented in the direction of the applied load when the tensile stress exceeds the tensile strength of concrete. Such cracks would be become a stochastic issue, in which the specimen shape and size are strength affecting parameters, as it is more probable to have a higher quantity of critical cracks in the bigger specimen, ultimately initiating specimen failure [3]. The insufficient energy is released at cracking onset for crack propagation in more ductile materials, or larger pores may block it, demanding additional energy for its cracking. A crack in concrete appears when the tensile stress of the concrete exceeds the tensile strength of concrete. Therefore, the tensile strength of concrete is of great importance, especially in the case where the main ingredients of concrete are changed. Splitting-tensile strength is the indirect measure of the tensile strength of concrete and is affected by many factors. The addition of dispersed short-discrete fibers to concrete increased the crack resistance and improved the mechanical characteristics [4,5,6,7,8,9,10,11,12]. Steel fibers are also employed to increase the toughness and post-cracking behavior of the cementitious material [13,14,15,16]. The concrete splitting-tensile strength is also affected by the size of the aggregates. The splitting-tensile strength would be higher in the case of finer aggregates because of their higher surface area, but with reduced bond stress between the aggregate and cement paste [17]. Usually, a lesser splitting-tensile strength was observed in the case of recycled aggregate concrete (RAC) in comparison to that of the respective natural aggregate concrete (NAC). The difference magnitude was based on multiple parameters relating to the RCA utilization. The higher replacement ratio of RCA would result in a reduced splitting-tensile strength, as frequently reported in the literature [18,19,20,21,22,23,24,25]. The behavior of RAC was more or less the same in terms of the splitting-tensile strength as in the case of compressive strength.

RCA has comparatively more porosity and water absorption and lower density and strength than the NCA [26,27]. Accordingly, the RAC has compromised mechanical characteristics and workability compared to that of NAC. Yang, et al. [28] reported a decrement in the compressive and splitting-tensile strength with an enhancement in the replacement ratio of the RCA. Hence, consideration should be given to the interrelation between the mix design and RAC mechanical characteristics prior to the construction. Multiple mathematical, empirical modeling has been conducted for the mechanical characteristics of RAC [29]; however, the said models were based on only limited input parameters and a small testing database calibration of models. In the urge to resolve this issue, artificial intelligence (AI) techniques are increasingly applied to foresee the mechanical characteristics of concrete. The highly precise modeling among the input and output parameters can be conducted by using AI techniques [30]. Due to advances in AI, the mechanical characteristics of concrete and other structural members may now be predicted using machine learning (ML) and other methods [31,32,33,34,35,36]. ML approaches (i.e., classification, regression, and clustering) are deployed for the statistical process and prediction of compressive strength with high accuracy in materials science and other fields [37,38,39,40,41,42]. High calcium fly ash geopolymer composite (FA-GPC) performance prediction by using ML techniques have previously been studied. Decision tree (DT), bagging, and adaptive boosting (AdaBoost) approaches were used to predict the strength of the FA-GPC. The error distribution process, model validation, regression, sensitivity analysis, and statistical checks were involved as a research objective to compare and confirm the employed algorithms’ accuracy. The civil engineering field can profit from the experimental cost savings, effort, and time by predicting the static properties of concrete with the help of ML approaches. The accuracy of prediction can be enhanced by integrating standalone models with an ensemble machine learning (EML) model, as depicted by other fields of study [43]. The employment of ensemble learning to predict concrete parameters has still only been studied with a limited scope. Random forest (random Forest) and adaptive boosting (AdaBoost) are the EML techniques that can enhance the prediction accuracy with the combination of voting and various regression tree forecasting on the ultimate result [44]. Song, et al. [45] determined the compressive strength of ceramic waste-modified concrete experimentally and with standalone techniques. The marginal variation in the experimental results and the prediction model outcomes were reported. Ahmad, et al. [44] performed EML and standalone techniques to predict the concrete’s compressive strength and accuracy of the comparison. It has been reported that the outcome predicted from the EML techniques had more precision than that by the standalone technique. However, the range in the standalone technique results was also acceptable.

The optimization of the RAC mix can be attained with the help of developed AI models. In various studies, only the data regarding the concrete composite mix proportions are usually accounted for as input variables instead of performing other additional measurements. However, the knowledge on the effect of various raw materials on the final strength is still missing, pointing out a research gap. Accordingly, the main aim of the current study was to explore a reliable but simple method for the prediction and evaluation of the input parameter’s effect on the output. The recycled aggregate concrete was explored by applying artificial intelligence, as presented in the current study. This study is important for understanding the significance of input parameters and their correctness in the ML algorithm results. Each model’s performance was additionally assessed using the k-fold cross-validation and statistical tests. Furthermore, to better understand the impacts/influences and interactions of the considered features, the post hoc model-agnostic method named as the SHAP analysis was also conducted. In addition, the post hoc model-agnostic method named as the SHapley Additive exPlanations (SHAP) may also be applied to have an insight into the ML models. Its performance on the existing artificial intelligence models is claimed to be the novelty of the current research. This would enhance the usage potential of the artificial intelligence methods for civil engineers in the construction industry. However, in terms of cost and time, the step-by-step laboratory process, which includes casting specimens, curing for a set period, and testing, remains a subject of concern. To tackle the challenges described above such as the excessive consumption of time and money, novel machine learning algorithms are being presented to anticipate the behavior of raw materials on the final output of concrete in terms of the splitting-tensile strength. This research aims to evaluate the effect of raw ingredients on the splitting-tensile strength of the RAC and its estimation using an artificial intelligence approach. Therefore, a precise forecasting model can be beneficial for researchers and engineers to assess the RAC mechanical characteristics and can conserve the cost and time in lieu of laboratory experimentations. Overall, we hope that this work will help explain the trends of machine learning approaches and how they can be used in different real-world domains to predict the strength properties of the concrete. We also hope that it will serve as a point of reference for both academics and professionals around the world, especially from a technical point of view.

## 2. Methods

### 2.1. Data Description

**Figure 1:**
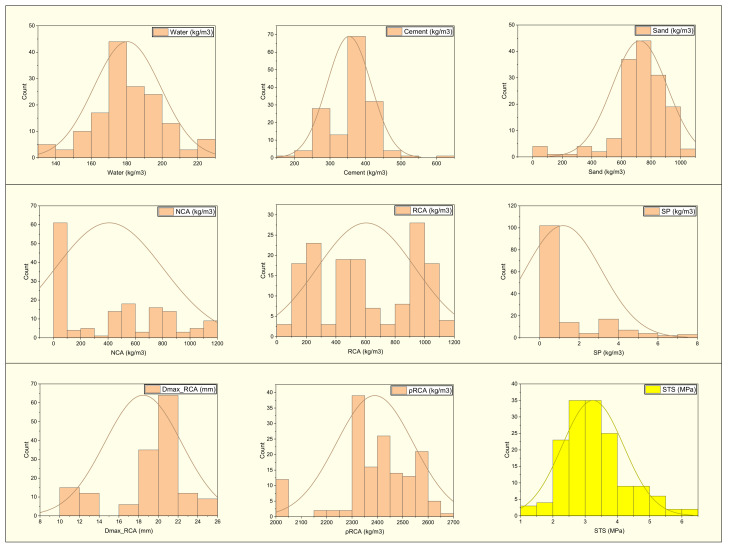
The distribution of the input and output parameters.

**Figure 2:**
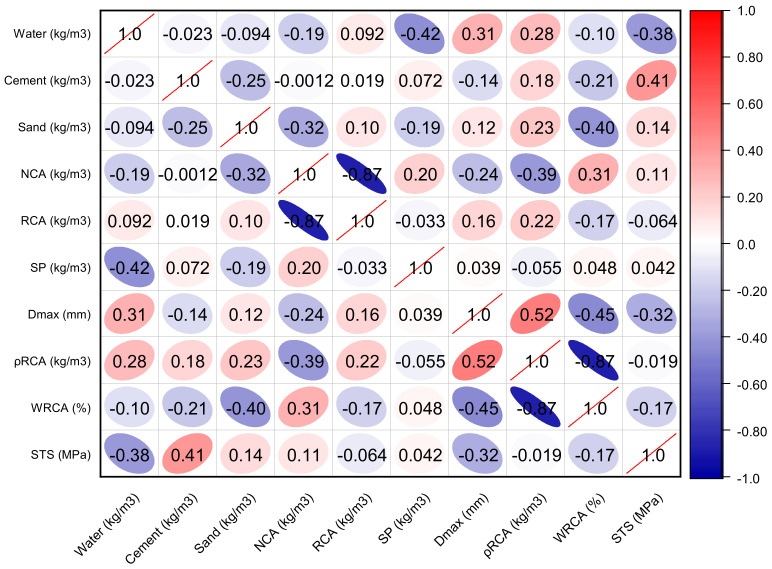
The correlation plot with the splitting-tensile strength.

The dataset comprises nine inputs: water, cement, sand, natural coarse aggregate (NCA), recycled coarse aggregate (RCA), superplasticizer (SP), maximum RCA size (Dmax_RCA), the density of recycled aggregate (ρRCA), and the water absorption of the recycled aggregate (WRCA). One output parameter was considered (i.e., the splitting-tensile strength (STS). A total of 154 mixes were collected for the splitting-tensile strength from 20 published experimental studies. Table 1 describes the statistical analysis of the input parameters with the splitting-tensile strength. The findings of the descriptive analysis are dependent on many input factors. Table 1 provides the lowest and maximum values and ranges for each variable utilized in the model. Other analytical parameters used to show the relevant values include the standard deviation, mean, mode, and a total of all data points for each variable. Figure 1 depicts the distribution of each component used in the mixes. Figure 2 depicts the correlation plot. In this scenario, no multicollinearity issues would be caused due to microscopic differences. If there is multicollinearity issues, then there is a strong effect of the input parameter between them, which ultimately affects the output results and may provide accurate findings. 

### 2.2. Machine Learning Models

**Figure 3:**
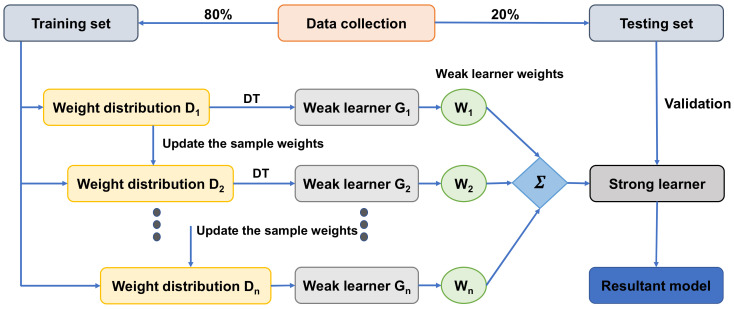
The complete process of prediction via the AdaBoost algorithm [47].

Figure 3 shows the entire process of forecasting the AdaBoost algorithm outcome. The ensemble technique is a concept of ML that is utilized to train various models by using a learning algorithm of the same kind [46]. Multiple algorithms are collected as multi-classifiers to make an ensemble. A group comprises of almost a thousand learners working with the same objective of resolving the issue. Ensemble learning is employed by an AdaBoost algorithm, which is a supervised ML technique. It can also be referred to as adaptive boosting, as the weights are re-linked to every instance, with higher weights linked to wrongly classified instances. Boosting techniques are widely utilized to minimize variance and bias in supervised ML. Weak learners can be strengthened by using the said ensemble techniques. An infinite number of DTs was employed for the input data during the training phase. During the construction of the initial DT, the erroneously categorized recorded data were given more priority throughout the initial model. The same data records were used only as the input for the other different models. The above-mentioned technique would be repeated until the creation of the specified base learners. AdaBoost optimizes the enhancement of the performance of DTs on binary classification issues. In addition, it is also used for enhancing the ML algorithm’s performance. It is specifically effective when it is used with slow learners. These ensemble algorithms are very prevalent in the civil engineering field, especially in predicting the mechanical properties of concrete.

**Figure 4:**
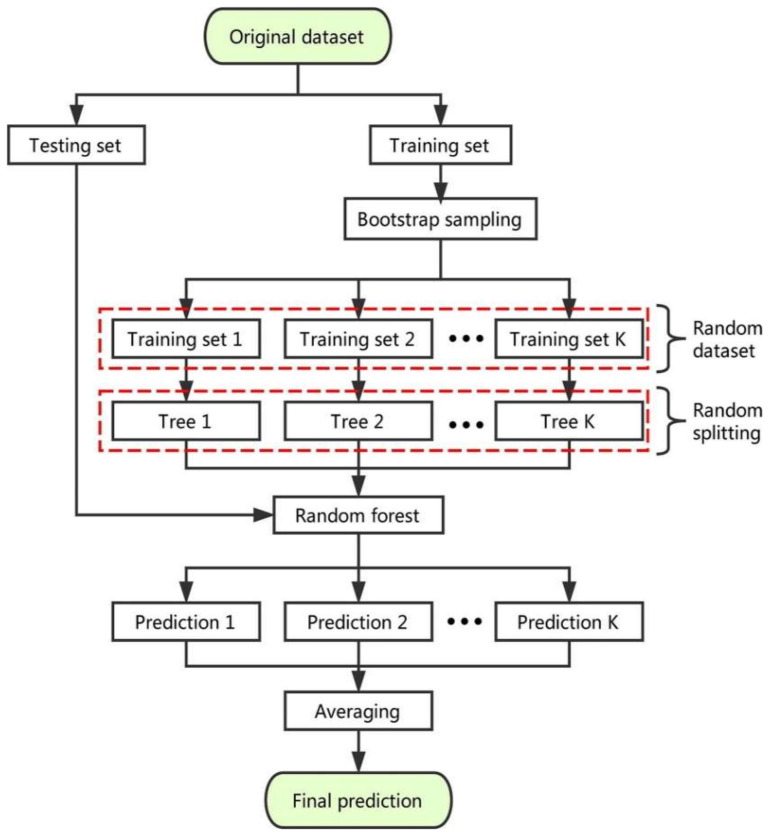
The random forest schematic diagram [48].

The random forest model is a regression and classification-based approach that has been studied by various researchers up to now [48,49]. The splitting-tensile strength of concrete was predicted using the random forest model, as conducted by Shaqadan [50]. The prime difference between random forest and DT was the number of trees, as shown in Figure 4. A single tree is developed in DT; however, in random forest, multiple trees are built that are known as forests. The dissimilar data are selected arbitrarily and accordingly allocated to respective trees. Each tree has data in rows and columns, and different dimensions of rows and columns are selected. The following steps were carried out for the growth of each tree; the data frame comprised two thirds of the whole data that were randomly selected for each tree. This method is known as bagging. Random selection was made for the prediction variables, and the node splitting was conducted by finely splitting these variables. For all trees, the remaining data were utilized to estimate the out-of-bag error. Accordingly, the final out-of-bag error rate was assessed by combining errors from each tree. Each tree provides regression, and among all of the forest trees, the forest with greater votes is selected for the model. The value of votes can either be 1s or 0s. The obtained proportion of 1s specifies the prediction probability. Random forest (random forest) is the most sophisticated among all of the ensemble algorithms. It includes desirable features for variable importance measures (VIMs) with robust overfitting resistance and fewer model parameters. DT is used as a base predictor for random forest. Acceptable results can be produced by random forest models with default parameter settings [51]. As allowed by random forest, combinations of parameter settings and base predictors can be reduced to one. Ensemble machine learning techniques were employed to achieve the objectives of this study on a conventional workstation by using Python coding via the software named Anaconda Navigator. The AdaBoost and random forest models were chosen in the software known as Spyder (Version 4.3.5), USA. Typically, these types of algorithms are built functions in the software and are employed using Python coding to forecast the needed results depending on the input parameters.

## 3. Results and Discussion

### 3.1. AdaBoost

**Figure 5:**
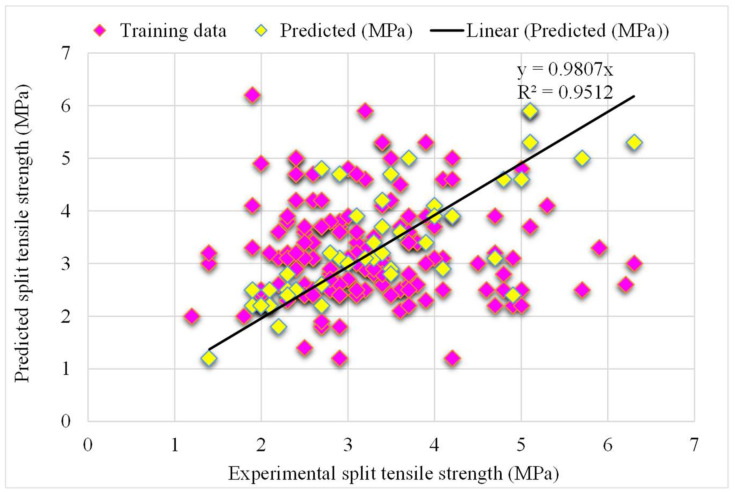
The AdaBoost model’s experimental and predicted results of the splitting-tensile strength.

**Figure 6:**
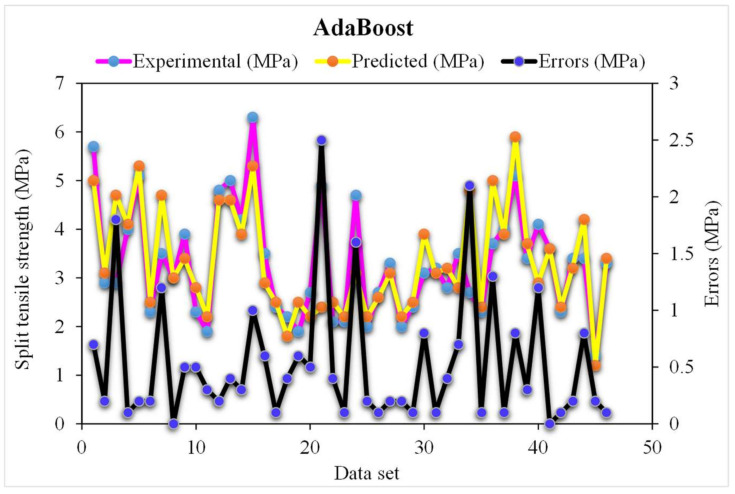
The AdaBoost model’s experimental and predicted values of the splitting-tensile strength with the errors.

A comparison of the projected and actual outputs of the AdaBoost model considering the experimental and predicted results is shown in Figure 5. The R^2^ value was 0.95, which showed better outcomes and could be used for the prediction of the splitting-tensile strength. The dispersion of the actual and predicted values and errors for the AdaBoost model is illustrated in Figure 6. However, 61% of the error values were below 0.5 MPa, 24% ranged from 0.5 to 1 MPa, and only 15% were higher than 1 MPa. Lower error values also represented the higher accuracy of the AdaBoost model as the maximum number of values presented the lower error rate between the experimental and predicted values. This ultimately shows the good predictive behavior of the AdaBoost model in terms of the splitting-tensile strength compared to the experimental data.

### 3.2. Random Forest Results

**Figure 7:**
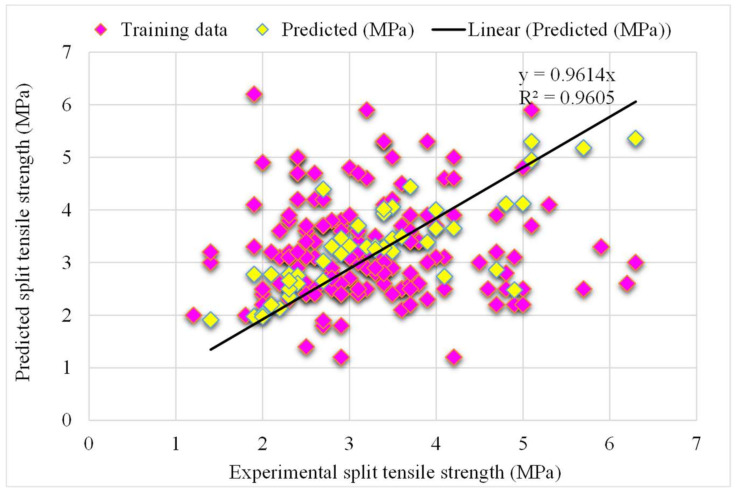
The random forest model’s experimental and the predicted splitting-tensile strength results.

**Figure 8:**
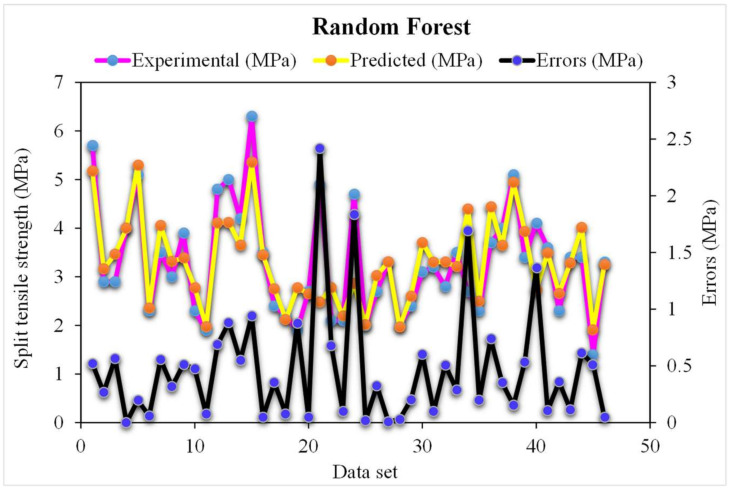
The random forest model’s experimental and predicted values of the splitting-tensile strength with the errors.

The correlation between the projected and actual results of the random forest model is shown in Figure 7. The R^2^ value for the random forest model was 0.96, which represents the highly precise and more accurate random forest with respect to the AdaBoost models. Furthermore, the dispersion of projected values, actual targeted values, and errors for the random forest model is shown in Figure 8. It was noted that 61% of the error data was below 0.5 MPa, 30% was from 0.5 to 1 MPa, and only 8% was higher than 1 MPa. Previous studies also reported the better performance of the random forest model in forecasting the various properties of different materials in terms of superior R^2^ and lower error values [47,52,53]. This analysis revealed a higher accuracy of the random forest model with respect to the AdaBoost models. It can also be depicted from the lower error and greater R^2^ values. In addition, twenty sub-models were employed by the AdaBoost and random forest to obtain the optimized value that produces a firm output.

### 3.3. Support Vector Machine (SVM) Results

**Figure 9:**
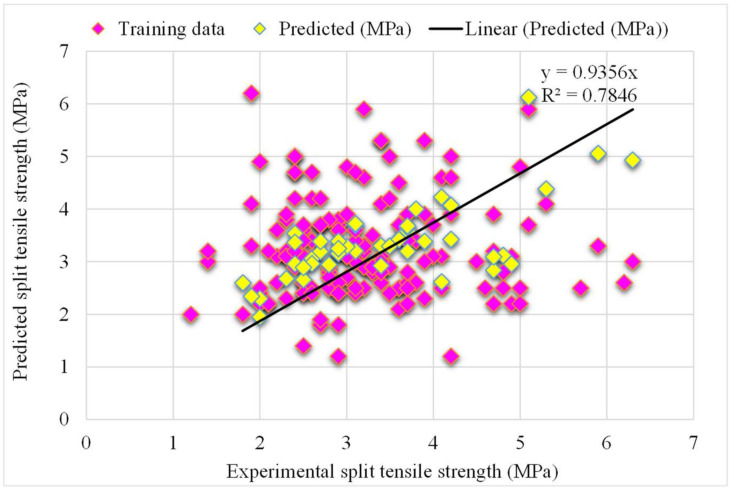
The SVM model’s experimental and predicted results of the splitting-tensile strength.

**Figure 10:**
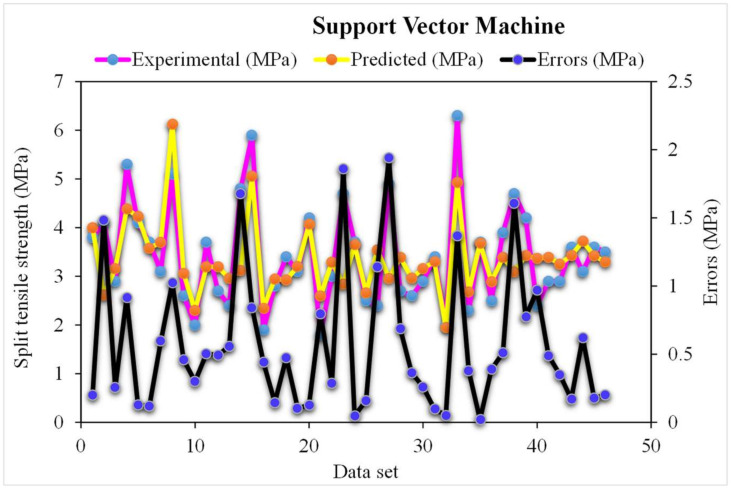
The SVM model’s experimental and predicted values of the splitting-tensile strength with the errors.

A comparison of the projected and actual outputs of the SVM model considering the experimental and predicted results is shown in Figure 9. The R^2^ value was 0.78, which showed better outcomes and could be used for the prediction of the splitting-tensile strength. The dispersion of the actual and predicted values along with errors for the SVM model, is illustrated in Figure 10. However, 52% of error values were below 0.5 MPa, 13% ranged from 0.5 to 1 MPa, and only 9% were higher than 1 MPa. The lower accuracy of the SVM model was also depicted by higher error values as the maximum number of values presented the higher error rate between the experimental and predicted values. This ultimately shows the poor predictive behavior of the SVM model in terms of the splitting-tensile strength compared to the results of the experimental data and other ensemble models.

### 3.4. Bagging Results

**Figure 11:**
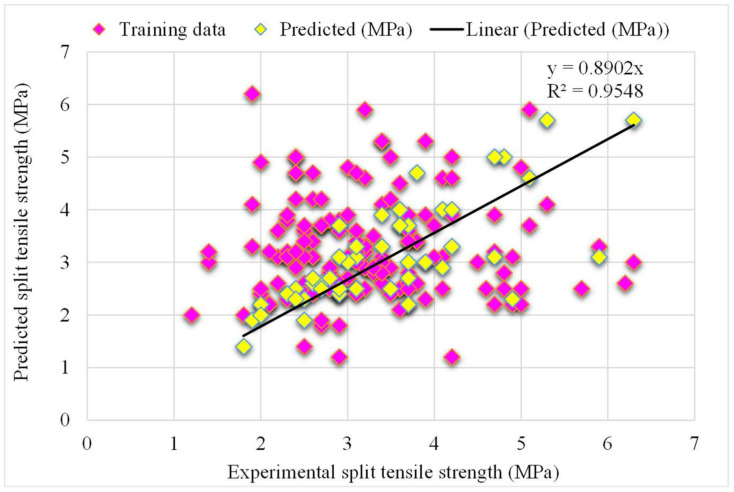
The bagging model’s experimental and predicted splitting-tensile strength results.

**Figure 12:**
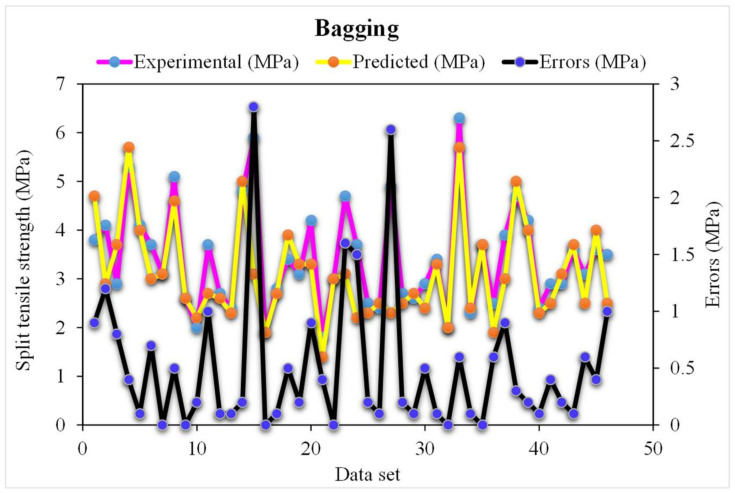
The bagging model’s experimental and predicted values of splitting tensile strength with the errors.

The correlation between the projected and actual results of the bagging model is shown in Figure 11. The R^2^ value for the bagging model was 0.95, which represents a highly precise and more accurate bagging with respect to the SVM models. Furthermore, the dispersion of the projected values, actual targeted values, and errors for the bagging model is shown in Figure 12. It was noted that 60% of the error data was below 0.5 MPa, 27% ranged from 0.5 to 1 MPa, and only 13% was higher than 1 MPa. Wang, et al. [47] reported that the AdaBoost machine learning approaches predicted the better compressive strength of geopolymer composites. Zhu, et al. [54] used the machine learning to forecast the splitting-tensile strength (STS) of the concrete containing recycled aggregate (RA) and revealed that the precision level of the bagging model was better. Ahmad, et al. [55] studied the boosting and AdaBoost ML approaches to predict the compressive strength of the high calcium fly-ash-based geopolymer. The bagging indicates better results. Previous studies have also reported the better performance of the random forest model in forecasting the various properties of different materials in terms of superior R^2^ and lower error values [47,52,53]. A higher accuracy of the random forest model with respect to the bagging models was revealed from this analysis. This was also depicted in the lower error and greater R^2^ values. In addition, twenty sub-models were employed by AdaBoost, random forest, and bagging to obtain the optimized value that produces a firm output.

### 3.5. K-Fold Cross-Validation Checks

**Figure 13:**
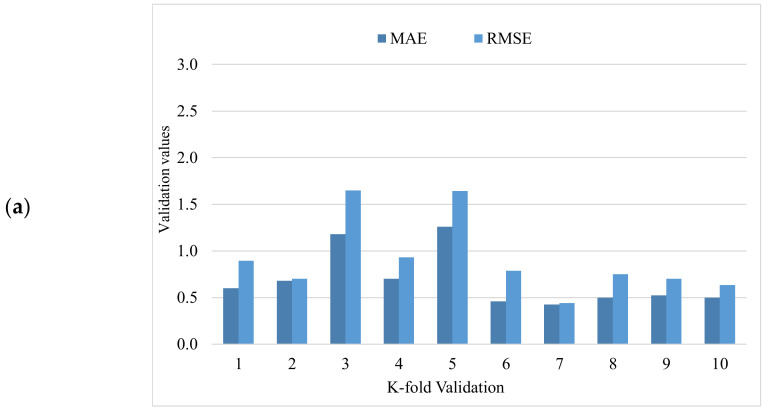

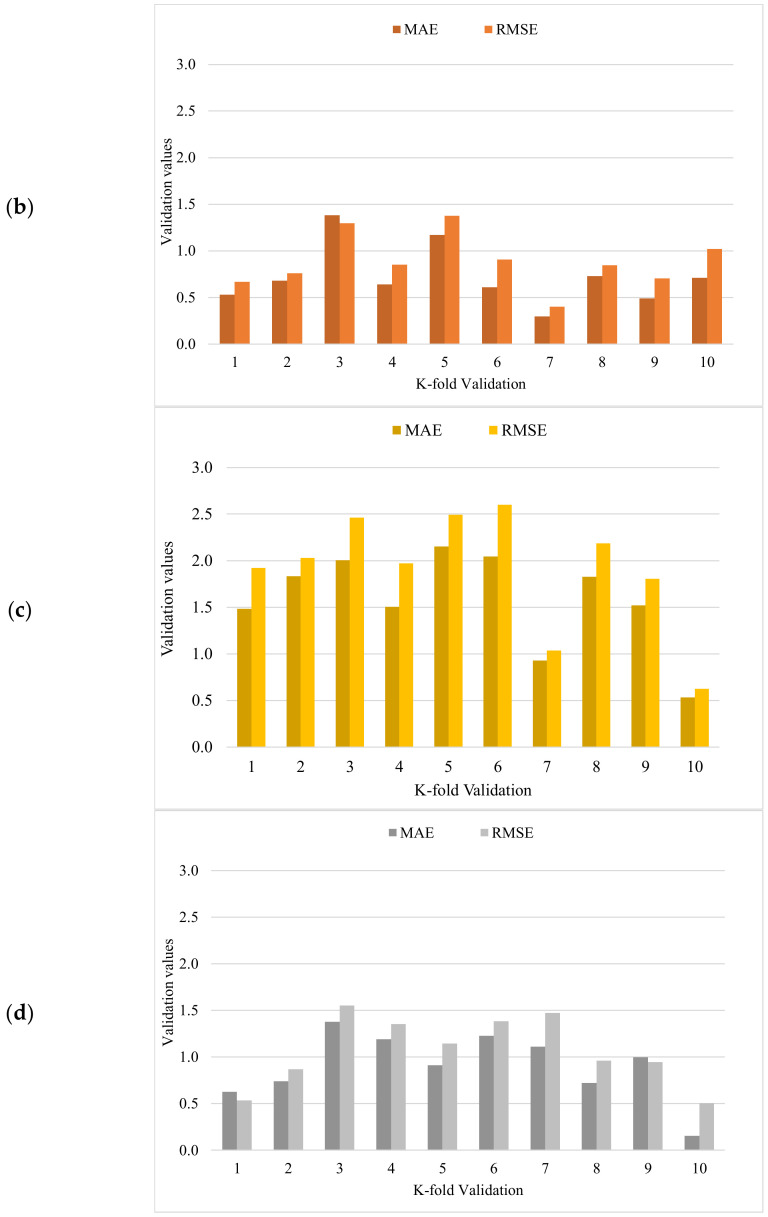
The k-fold cross-validation: (**a**) AdaBoost model; (**b**) random forest model; (**c**) SVM model; (**d**) B = bagging model.

Statistical analysis with Equations (1) and (2) was utilized to predict the model’s response. Statistical checks were used to evaluate the performance of the models [44,52,56,57]. The model’s legitimacy was evaluated by utilizing the k-fold cross-validation approach during execution. Usually, the model’s validity is conducted with a k-fold cross-validation process [58], in which random dispersion is carried out by splitting it into ten groups. A total of 70% of data was used as the training data and 30% was used for testing. The R2 for all training datasets was greater than 0.85. The greater the R^2^ value and lower the errors (RMSE and MAE), the more accurate the model is. Furthermore, this process should be repeated multiple (i.e., 10) times for a satisfactory result. The exceptional precision of the model can be achieved by using this comprehensive approach. In addition, statistical analysis (i.e., RMSE and MSE) was also performed for all of the models (Table 2). The random forest model’s accuracy (inversely related to error values) compared to the AdaBoost models was also supported by these checks. Statistical analysis, as reported in the literature [37,59], has been used to assess the model’s response to prediction. The k-fold cross-validation was evaluated by utilizing R^2^, RMSE, and MAE. Respective dispersions for the random forest and AdaBoost models are presented in Figure 13. The average and maximum values of R^2^ for AdaBoost were 0.72 and 0.95, respectively (Figure 13a). The maximum and average values of R^2^ for the random forest model were 0.96 and 0.77, respectively, are shown in Figure 13b. Upon comparing the error values (RMSE and MAE), the RMSE and MAE values for all models are shown in Table 2. The random forest model with the lowest error and higher R^2^ value performed best for the results prediction.

(1) $$\mathrm{MAE}=\frac{1}{n}\sum_{i=1}^{n}\left|x_{i}-x\right|$$

(2) $$\mathrm{RMSE}=\sqrt{\sum \frac{{\left(y_{pred}-y_{ref}\right)}^{2}}{N}}$$

where:

*n* = Total data samples,

x, $y_{ref}$ = Data sample reference values,

$x_{i}$, $y_{pred}$ = Model prediction values.

**Table 2:** The statistical description of all models.

Models	MAE (MPa)	RMSE (MPa)	R^2^
AdaBoost	0.53	0.60	0.95
Random Forest	0.48	0.49	0.96
SVM	0.85	0.95	0.78
Bagging	0.51	0.64	0.95

### 3.6. Parameter Tuning for Ensemble Learner

**Figure 14:**
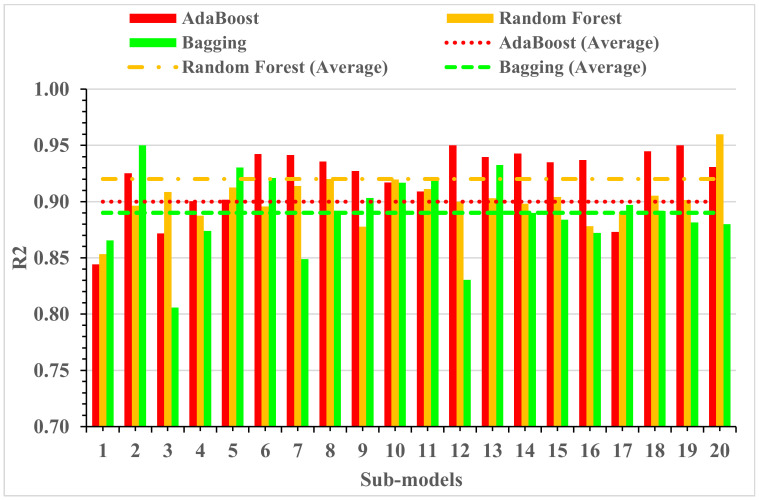
The R^2^ values of the sub-models.

The learning rates and other features that specifically effect ensemble approaches can be used as tuning parameters for the models used in ensemble techniques. Boosting ensemble models (20 each) with 10, 20, 30,…, 200 component sub-models were developed for base learners in this work, and the correlation with high coefficient values was utilized to identify the best model. The ensemble model accuracy and the number of component sub-models is shown in Figure 14. The ensemble AdaBoost, random forest, and bagging models with 12, 20, and 2 or more sub-models demonstrated a significant result with a better R^2^ value. Initially, the analysis revealed that utilizing ensemble modeling enhanced the accuracy of both models. However, for the random forest ML approach, using 200 component sub-models while 12 or more for the AdaBoost algorithms and two or more sub-models for bagging, provided more accurate results. The architectures employed in the ensemble models are described in Table 3. It is worth mentioning that the learning rate was 0.95, 0.95 and 0.96 when AdaBoost, bagging, and random forest algorithms were applied. However, the SVM, being an individual algorithm, presented unacceptable results. 

The ensembled ML and individual approaches were explored in this study to estimate the RAC. Random forest and AdaBoost machine learning techniques were used in this study to predict the splitting-tensile strength of the RAC. To establish the algorithm’s prediction superiority, the employed algorithms were compared for the targeted performance. The output of the random forest model was more accurate, having a 0.96 R^2^ value compared to AdaBoost with 0.95 R^2^ and bagging with 0.95. Furthermore, the performance of the AdaBoost and random forest models was also evaluated by utilizing the k-fold cross-validation technique and statistical analysis. The performance of the model was higher with low error levels. However, it is tough to assess the optimized machine learning regressors to forecast results from a wide range of topics because the model’s performance is very much dependent on the data points and the model’s input parameters. On the other hand, in ensemble ML techniques, sub-models are generated to leverage the weak learner that can be optimized and trained on data to achieve a higher value of R^2^. The dispersion of values for the determinant coefficient of the bagging, AdaBoost, and random forest sub-models is shown in Figure 11. The values of R^2^ for all sub-models of random forest were greater than 0.85, while most values of R^2^ in the case of sub-models for AdaBoost were less than 0.84, respectively. It depicts a higher accuracy of the random forest technique for the results prediction, having a maximum value of R^2^ (i.e., 0.96). Therefore, the random forest model is suggested to predict the splitting-tensile strength of RAC.

The randomization technique further revealed the statistical importance. The test was carried out by (1) permuting the dataset’s activity values repeatedly, (2) generating RF models from the permuted values, and (3) comparing the resulting scores to the score of the original RF model derived from non-randomized activity values. If the original RF model is statistically significant, the score from permuted data should be significantly higher. Figure S1 shows the R^2^ values for 30 trials based on the permuted data. The original model’s R^2^ value was substantially greater than any of the permuted data trials. As a result, the RF model was statistically significant and reliable.

### 3.7. Enhanced Explainability of the ML Models

**Figure 15:**
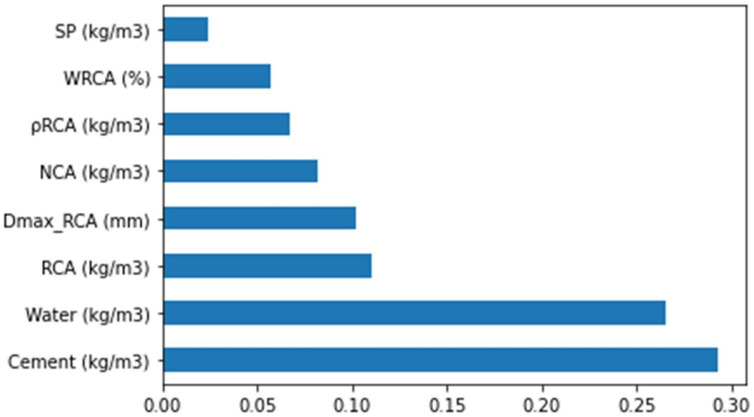
The feature importance of the input parameters.

This study provides a detailed explanation of the machine learning model. Furthermore, the dependencies of the corresponding features were also explored. The SHAP tree explainer was initially implemented over the whole database to provide an enhanced description of the global feature influences by merging the local SHAP explanations. The employment of a tree-like SHAP approximation method named as “TreeExplainer” was conducted [60]. In this method, the evaluation of the internal structure of the tree-based models (i.e., summation for a set of calculations linked with the tree model leaf node), which led to the complexity of low-order, was conducted [60]. It was observed that the random forest model had a highly precise prediction performance for the splitting-tensile strength of the RAC. Therefore, in this section, the interpretation of the model was conducted for the splitting-tensile strength of the RAC by applying SHAP. The SHAP value correlation of the different considered features for the splitting-tensile strength of the RAC (as attained from the ensembled random forest modeling) is shown in Figure 15.

This assessment was based on a database implied in the current research, and the outcomes with a higher precision may also be obtained in the case of more data points. It may be noted here that the cement content had the highest feature value of 0.29 in the case of the STS prediction for the RAC, as cement is a key feature of strength development. The water content feature value was 0.26 (i.e., a key parameter in the case of RAC due to a greater water absorption capability of the recycled aggregates), depicting the second highest SHAP value. Subsequently, the content of RCA was the third most influencing factor with a feature value of 0.11, as shown in Figure 12. The RCA content directly influences the strength of the RAC and the available water–cement ratio. Similarly, it had more or less the same influence as for the RCA maximum size as the aggregate size has a significant role in the strength development of concrete. Natural coarse aggregate (NCA) content was ranked fourth for having a higher SHAP value. As in the case of the RAC, the RCAs were used to replace the NCAs; therefore, the NCA content feature has considerable influence in terms of the RAC strength development. Similarly, the influence of RCA density was next in terms of the SHAP value, followed by the water absorption of RCA and content of the superplasticizer features. All of these features have their unique roles in the splitting tensile strength of RAC.

**Figure 16:**
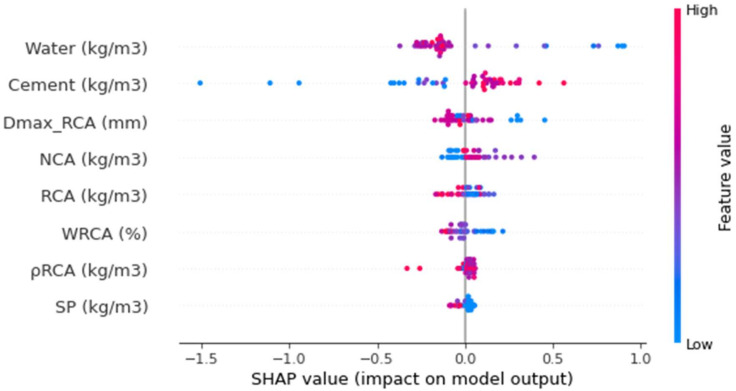
The SHAP plot of the input parameters.

Figure 16 shows the values on the violin SHAP plot for all of the corresponding features that are taken to forecast the splitting-tensile strength of the RAC. Each feature value is represented by a unique color in this plot, and the respective SHAP value at the x-axis shows the contribution output. As an illustration, the water content is an input feature with a higher influence (Figure 16) but has a negative influence in depicting the inverse relationship for this feature with the splitting-tensile strength of the RAC. It means that increasing the water content would decrease the strength and vice versa. A SHAP value of almost 1 in the form of blue points (low-value color) at the rightmost showed that the higher water content decreased the RAC splitting-tensile strength. However, in the case of the cement feature, a positive influence was seen. The RCA particle size also influences the RAC STS both positively and negatively. A very much larger particle size would also affect the RAC strength. In the same manner, the RCA influences both negatively and positively. The RCA content up to the optimum content was a positive influence, while a negative influence was observed beyond this content. Afterward, the RCA water absorption was negatively influenced as this water absorption ability would absorb more water, leaving a compromised w/c ratio for the hydration process, ultimately affecting the concrete strength development process. Likewise, the RCA density and superplasticizer were also on the borderline and both had positive and negative influences depending on the optimum content.

**Figure 17:**
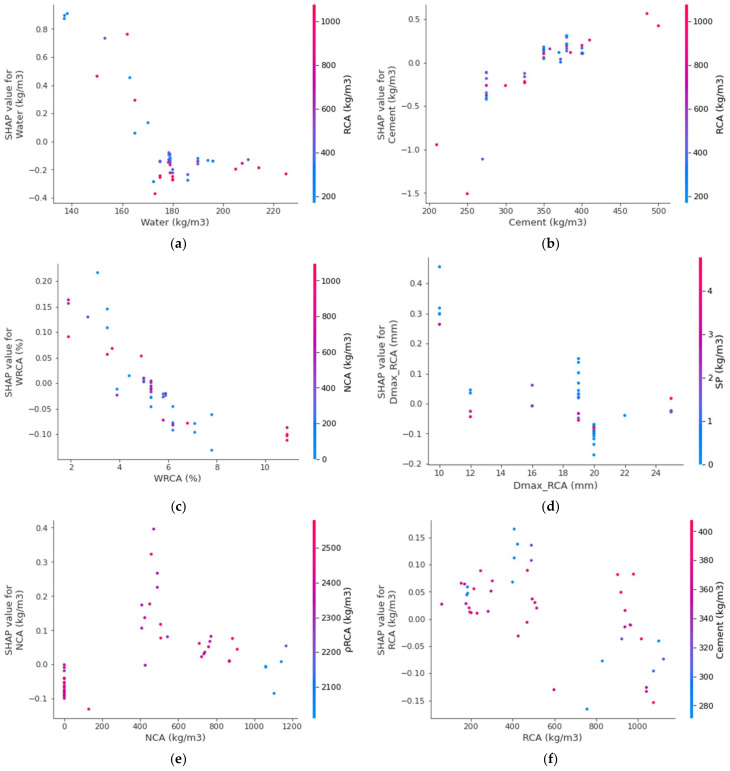
The interaction plot of various parameters: (**a**) Water; (**b**) Cement; (**c**) WRCA; (**d**) Dmax_RCA; (**e**) NCA; (**f**) RCA.

The interaction of the features with the RAC splitting-tensile strength is provided in Figure 17. The water feature interaction is shown in Figure 17a. It can be observed from the plot that the water mainly interacted with the RCA and had a negative/inverse relationship out of the many factors. As in this scenario, the RCAs’ higher water absorption ability leaves a compromised water content for the hydration process, hence impacting the strength development process of concrete. In Figure 17b, the positive influence of cement on the splitting-tensile strength of RAC is observed. More interaction of cement occurs with the RCA content and is directly related. The RCA water absorption feature interaction is plotted in Figure 17c. The WRCA indicates the negative impact due to its effect on the w/c ratio required for the hydration process to develop concrete strength. Therefore, the said effect would result in a decreased splitting-tensile strength. Then, the RCA particle size showed both positive and negative impacts, and had more interaction with the superplasticizer (Figure 17d). This might be due to the effect of the surface-area requirements on the w/c ratio. The interaction of NCA is shown in Figure 17e, depicting both positive and negative impacts depending upon the optimum content. Up to the optimum content, the lesser content would end up with a positive interaction and vice versa. Similarly, RCA was first positively and then negatively interacting based on the optimum content (Figure 17f).

## 4. Conclusions

This paper estimated the splitting-tensile strength of recycled coarse aggregate (RAC) using artificial intelligence algorithms. Additionally, the effect of raw materials on the splitting-tensile strength was studied, and their interactions discussed. Based on the conducted research, the following conclusions were drawn:

1. The AdaBoost, bagging, and random forest models had R^2^ values of 0.95, 0.95, and 0.96, respectively. However, the ensemble model results for random forest, followed by AdaBoost and bagging were acceptable. On the other hand, the SVM model with an R^2^ of 0.78 presented unacceptable results. Due to its greater R^2^ and lower error levels, the random forest model outperformed AdaBoost, bagging, and SVM techniques in terms of prediction.
2. The k-fold cross-validation technique and statistical analysis revealed satisfactory random forest, bagging, and AdaBoost outcomes. The random forest model’s lower MAE value of 0.48 MPa also showed that it outperformed the AdaBoost models with a MAE of 0.53 MPa.
3. The lower RMSE error of 0.49 MPa for the random forest model in this study validates the application of machine learning to forecast the splitting-tensile strength of the RAC and their raw material effect. However, the RMSE error of the AdaBoost and bagging was 0.60 and 0.64, respectively. On the other hand, the RSME error of the SVM was 0.95 with unsatisfactory results.
4. The presented techniques using artificial intelligence seem reliable for predicting the interaction of raw ingredients on the splitting-tensile strength of the recycled aggregate concrete.
5. The cement content had the highest impact on the RAC splitting-tensile strength prediction, followed by the water content, as depicted from the SHAP analysis. However, the superplasticizer content feature was the least influencing on the splitting-tensile strength of the RAC.
6. The feature interaction plot showed that the water content had a negative correlation, whereas the cement content positively influenced the RAC splitting-tensile strength. Furthermore, the main interaction of cement is with water. A higher SHAP plot value in the form of blue points (lower value color) depicts the inverse relation of water content with the RAC splitting-tensile strength.

It is recommended that more thorough research on recycled aggregate concrete should carried out by considering more input and output parameters. Increasing the number of input variables and expanding the database can result in more reliable results and a more comprehensive interpretation. The compressive strength, temperature effect, acid attack resistance, chloride resistance, sulfate resistance, and corrosion should all be included in the future. To produce more accurate predictions, advanced technologies such as particle swarm optimization (PSO) and M5P tree can be applied. Machine learning approaches can be used with heuristic methods such as the whale optimization algorithm and ant colony optimization for effective outcomes, which can then be compared to the current study.

## Figures and Tables

**Table 1:** The details of the input and output data.

	Mean	Standard Error	Median	Mode	Standard Deviation	Range	Minimum	Maximum
Water (kg/m^3^)	180.4	1.5	179.0	179.0	18.9	88.0	137.0	225.0
Cement (kg/m^3^)	353.7	5.0	372.0	380.0	62.2	442.0	158.0	600.0
Sand (kg/m^3^)	723.7	15.1	730.0	927.0	186.6	1010.0	0.0	1010.0
NCA (kg/m^3^)	407.7	31.8	443.7	0.0	393.8	1168.0	0.0	1168.0
RCA (kg/m^3^)	604.5	26.7	538.0	970.0	330.2	1066.0	57.0	1123.0
SP (kg/m^3^)	1.2	0.2	0.0	0.0	1.9	7.8	0.0	7.8
*D_max_RCA_* (mm)	18.5	0.3	20.0	20.0	3.9	15.0	10.0	25.0
*ρ*_RCA_ (kg/m^3^)	2382.3	12.8	2390.0	2320.0	153.4	651.0	2010.0	2661.0
W_RCA_ (%)	5.5	0.2	5.3	5.3	2.1	9.0	1.9	10.9
STS (MPa)	3.2	0.1	3.1	3.7	1.0	5.1	1.2	6.3

**Table 3:** The detailed information of the sub-models of the ensemble approaches.

Approach Used	Ensemble Techniques	Ensemble Techniques	Individual Techniques	Ensemble Techniques
Machine learning methods	AdaBoost	Random forest	Support vector machine (SVM)	Bagging
Ensembled models	(10, 20, 30,…, 200)	(10, 20, 30,…, 200)	-	(10, 20, 30,…, 200)
Optimum Estimator	12	20	-	02
R^2^ Value	0.95	0.96	0.78	0.95

## Data Availability

Not applicable.

## Supplementary Materials

The following supporting information can be downloaded at: https://www.mdpi.com/article/10.3390/ma15124194/s1, Table S1: Database for recycled aggregate concrete; Figure S1: RF randomization test [61,62,63,64,65,66,67,68,69,70,71,72,73,74,75,76,77,78].
