# Supplementary material for: Use of Artificial Intelligence Methods for Predicting the Strength of Recycled Aggregate Concrete and the Influence of Raw Ingredients
Source: Materials (Basel). 2022 Jun 13;15(12):4194. doi: 10.3390/ma15124194 (PMC9229192; doi:10.3390/ma15124194)
Supplement: Supplementary file 1 [file materials-15-04194-s001.zip › materials-1716357-supplementary.pdf]

Additional Information

1. AdaBoost

```
from sklearn.ensemble import AdaBoostRegressor

# Importing the datasamples

WM=pd.read_csv('STS.csv')

WM.head()

X=WM.iloc[:,:-1].values

y=WM.iloc[:,-1].values

# Splitting into train set and test set

from sklearn.model_selection import train_test_split

X_train, X_test, y_train, y_test = train_test_split(X, y, test_size = 0.3, random_state = 0 )
```

2. Random Forest

```
from sklearn.ensemble import BaggingRegressor,RandomForestRegressor

# Importing the datasamples

WM=pd.read_csv('STS.csv')

WM.head()

X=WM.iloc[:,:-1].values

y=WM.iloc[:,-1].values

from sklearn.model_selection import train_test_split

X_train, X_test, y_train, y_test = train_test_split(X, y, test_size = 0.30, random_state = 0 )
```

3. SVM

```
from sklearn.svm import SVM
# Importing the datasamples
WM=pd.read_csv('STS.csv')
WM.head()
X=WM.iloc[:,:-1].values
y=WM.iloc[:,-1].values
# Splitting into train set and test set
from sklearn.model_selection import train_test_split
X_train, X_test, y_train, y_test = train_test_split(X, y, test_size = 0.30, random_state = 0 )
```

4. Bagging

```
from sklearn.ensemble import BaggingRegressor
# Importing the datasamples
WM=pd.read_csv('STS.csv')
WM.head()
X=WM.iloc[:,:-1].values
y=WM.iloc[:,-1].values
# Splitting into train set and test set
from sklearn.model_selection import train_test_split
X_train, X_test, y_train, y_test = train_test_split(X, y, test_size = 0.30, random_state = 0 )
```

5. Dataset

Table S1. Database for recycled aggregate concrete.

Ref.	Water (kg/m^3)	Cement (kg/m^3)	Sand (kg/m^3)	NCA (kg/m^3)	RCA (kg/m^3)	SP (kg/m^3)	D_{max_RCA} (mm)	ρ_{RCA} (kg/m^3)	W_{RCA} (%)	STS (MPa)
[1]	207.6	400	662	863	153	0	20	2410	5.8	3.7
	207.6	400	662	697	298	0	20	2410	5.8	3.6
	207.6	400	662	383	573	0	20	2410	5.8	3.4
	207.6	400	662	0	903	0	20	2410	5.8	3.3
[2]	165	300	765	905	267	4.98	25	2430	4.4	3
	165	318	739	608	537	6.042	25	2430	4.4	3.2
	162	325	683	0	1123	6.175	25	2430	4.4	3.2
[3]	160.6	380	598	1182	52	4.9	20	2165	6.8	
	165.4	380	529	1175	103	4.9	20	2165	6.8	
	170.2	380	460	1168	154	4.9	20	2165	6.8	3.7
	175.6	380	327	1162	254	4.9	20	2165	6.8	
	180.9	380	0	1162	509	4.9	20	2165	6.8	3
[4]	180	400	708	886	215	0	20	2570	3.5	3.2
	180	400	708	554	538	0	20	2570	3.5	3
	180	400	708	0	1075	0	20	2570	3.5	2.8
	225	410	642	840	204	0	20	2570	3.5	3.2
	225	410	642	524	506	0	20	2570	3.5	3.2
	225	410	642	0	1017	0	20	2570	3.5	3.1
[5]	225	410	642	840	204	0	20	2580	3.5	3.6
	225	410	642	524	506	0	20	2580	3.5	3.4
	225	410	642	0	1017	0	20	2580	3.5	3.3

	205	410	662	865	210	0	20	2580	3.5	3.6
	205	410	662	541	525	0	20	2580	3.5	3.6
	205	410	662	0	1049	0	20	2580	3.5	3.4
	180	400	708	886	215	5.6	20	2580	3.5	3.7
	180	400	708	554	538	5.6	20	2580	3.5	3.7
	180	400	708	0	1075	5.6	20	2580	3.5	3.5
	160	400	729	912	221	7.8	20	2580	3.5	4.1
	160	400	729	570	554	7.8	20	2580	3.5	4
	160	400	729	0	1107	7.8	20	2580	3.5	3.8
[6]	175	350	730	711	297	1.68	25	2530	1.9	4.2
	175	350	730	508	494	1.68	25	2530	1.9	4
	175	350	730	0	989	1.68	25	2530	1.9	3.8
	175	350	730	711	282	1.68	25			3.6
	175	350	730	508	469	1.68	25	2400	6.2	3.4
	175	350	730	0	938	1.68	25	2400	6.2	3.2
[7]	186	372	617.65	772.67	515.55	0	20	2400		2.8
	186	372	617.65	515.11	772.67	0	20	2400		2.7
	186	372	617.65	257.56	1030.22	0	20	2400		2.2/
	186	372	494.12	128.78	123.53	0	20	2630		2.6
	186	372	370.59	128.78	247.06	0	20	2630		2.5
	186	372	247.06	128.78	370.59	0	20	2630		2.3
	186	372	123.53	128.78	494.12	0	20	2630		2.0
[8]	200	270	750	675	200	1.08	19	2440	5.8	1.9
	210	270	750	450	400	1.35	19	2440	5.8	1.9
	220	270	750	225	600	1.62	19	2440	5.8	1.4

	165	370	865	760	230	1.48	19	2440	5.8	3.1
	165	370	865	505	455	1.85	19	2440	5.8	3.1
	165	370	865	250	680	2.59	19	2440	5.8	2.9
[9]	178.5	275	938.05	723.07	180.77	1.925	16	2400	5	2.4
	178.5	275	962.73	423.77	423.77	1.925	16	2400	5	2.5
	178.5	275	1005.18	0	756.46	1.925	16	2400	5	2.6
	190	380	794.31	750.04	187.57	2.66	16	2400	5	3.1
	190	380	811.37	443.71	443.71	2.66	16	2400	5	2.9
	190	380	838.29	0	807.97	2.66	16	2400	5	2.9
[10]	172.43	401	574	911	303	0.2005	20	2661	1.9	2.3
	172.43	401	574	585	585	0.70175	20	2602	2.6	2.1
	172.43	401	574	0	1119	0.90225	20	2510	3.9	2
[11]	150	250	762	858	286	4.375	19			2.2
	150	250	753	564	564	4.375	19			1.8
	150	250	743	279	836	4.375	19			1.4
	150	250	734	0	1100	4.375	19			1.2
	180	400	685	770	257	3	19			3.1
	180	400	676	507	507	3	19			2.7
	180	400	667	250	751	3	19			2.5
	180	400	659	0	988	3	19			2.1
[12]	193	350	661	1061	57	0	12	2010	10.9	2.9
	194	350	515	1061	170	0	12	2010	10.9	2.7
	196	350	368	1061	283	0	12	2010	10.9	2.6
	199	158	0	1061	566	0	12	2010	10.9	2.5
	158	350	693	1111	59	3.5	12	2010	10.9	3.4

	163	350	536	1105	177	3.5	12	2010	10.9	3.3
	168	350	381	1100	294	3.5	12	2010	10.9	3.1
	178	350	0	1089	582	3.5	12	2010	10.9	3
	137	350	713	1143	61	3.5	12	2010	10.9	4.2
	139	350	555	1143	183	3.5	12	2010	10.9	4.5
	143	350	395	1138	304	3.5	12	2010	10.9	3.7
	150	350	0	1132	605	3.5	12	2010	10.9	3.4
[13]	180	281	802	0	970	0	10	2360	4.7	3.5
	170	293	648	0	919	0	10	2280	6.2	3.1
	165	337	841	0	879	0	10	2220	7.8	3.3
	190	463	621	0	970	0	10	2360	4.7	3.8
	190	500	621	0	919	3.24	10	2280	6.2	3.7
	180	600	567	0	879	5.04	10	2220	7.8	3.7
	220	537	693	782	138	0	20	2330	4.4	
[14]	179	275	878	735	184	0	20	2320	5.3	2.8
	179	275	849	455	455	0	20	2320	5.3	3.1
	179	275	868	0	830	0	20	2320	5.3	2.4
	190	380	744	757	189	0	20	2320	5.3	3.5
	190	380	710	471	471	0	20	2320	5.3	2.7
	190	380	715	0	874	0	20	2320	5.3	3.7
	179	275	961	740	185	0	20	2320	5.3	2.5
	179	275	978	408	408	0	20	2320	5.3	2.5
	179	275	1010	0	640	0	20	2320	5.3	2.3
	190	380	813	767	192	0	20	2320	5.3	2.8
	190	380	822	426	427	0	20	2320	5.3	2.6

	190	380	836	0	683	0	20	2320	5.3	2.3
	179	325	799	839	210	0	20	2320	5.3	2.8
	179	325	831	490	490	0	20	2320	5.3	2.7
	179	325	825	0	923	0	20	2320	5.3	2.3
	173	385	698	892	223	0	20	2320	5.3	3.1
	173	385	742	515	515	0	20	2320	5.3	3.9
	173	385	746	0	963	0	20	2320	5.3	2.4
	159.8	340	556	1020	238	0	20	2336	3.6	
[15]	159.8	340	556	638	596	0	20	2315	3.6	
	159.8	340	556	319	894	0	20	2295	3.6	
	137.1	380	927	869.2	202	0	10	2470	3.7	5.7
	146.5	380	927	543.2	505.1	0	10	2470	3.7	5.
	162.3	380	927	0	1010.2	0	10	2470	3.7	5.1
	138.2	380	927	869.2	195	0	10	2390	4.9	6.3
[16]	149.8	380	927	543.2	487.5	0	10	2390	4.9	5.1
	170.4	380	927	0	975.1	0	10	2390	4.9	5.9
	139.7	380	927	869.2	187.8	0	10	2300	5.9	5.3
	153.1	380	927	543.4	469.4	0	10	2300	5.9	6.2
	175	380	927	0	938.8	0	10	2300	5.9	4.2
	205	300	697	0	1075	0	20	2450	3.1	2.5
	205	300	697	0	1027	0	20	2370	7.1	2.4
[17]	205	300	697	0	1027	0	20	2360	7.8	1.9
	180	350	706	0	1089	0	20	2450	3.1	3.4
	180	350	706	0	1041	0	20	2370	7.1	2.6
	180	350	706	0	1041	0	20	2360	7.8	2.6

	185	425	696	0	1028	0	20	2450	3.1	3.9
	185	425	696	0	982	0	20	2370	7.1	3.7
	185	425	696	0	982	0	20	2360	7.8	3.4
	165	485	685	0	1039	0	20	2450	3.1	4.7
	165	485	685	0	979	0	20	2370	7.1	4.1
	165	485	685	0	982	0	20	2360	7.8	4.2
[18]	178.3	358	730.4	783.6	299.3	0.3	19	2570	2.7	3.9
	178.3	358	730.4	458.3	598.4	0.3	19	2570	2.7	3.9
	178.3	358	730.4	0	1020	0.3	19	2570	2.7	3.3
[19]	214.2	210	929	0	966	0	22	2451	7.8	2
	196	280	866	0	940	0	22	2387	6.9	2.9
	161	350	858	0	974	3.5	22	2362	4.2	4.6
	212.1	210	932	0	970	0	22	2456	7.5	2
	193.2	280	870	0	970	0	22	2455	6.4	2.9
	157.5	350	858	0	1029	3.5	22	2496	4.2	4.8
	207.9	210	938	0	953	0	22	2401	7.6	2.1
	187.6	280	877	0	988	0	22	2484	5.4	3
	150.5	350	868	0	982	3.5	22	2363	3.6	4.9
	205.8	210	943	0	977	0	22	2447	6.9	2.2
	190.4	280	873	0	962	0	22	2458	5.8	3
	157.5	350	858	0	1016	3.5	22	2464	3.9	5.0
[20]	179	275	878	735	184	0	19	2320	5.3	4.1
	179	275	849	455	455	0	19	2320	5.3	4.7
	179	275	868	0	830	0	19	2320	5.3	4.9
	190	380	714	757	189	0	19	2320	5.3	4.7

	190	380	710	471	471	0	19	2320	5.3	4.8
	190	380	715	0	874	0	19	2320	5.3	5.0
	179	275	961	740	185	0	19	2320	5.3	2.5
	179	275	978	408	408	0	19	2320	5.3	2.4
	179	275	1010	0	640	0	19	2320	5.3	2.3
	190	380	813	767	192	0	19	2320	5.3	3.2
	190	380	822	426	427	0	19	2320	5.3	2.7
	190	380	836	0	683	0	19	2320	5.3	2.4
	179	325	799	839	210	0	19	2320	5.3	2.9
	179	325	831	490	490	0	19	2320	5.3	2.6
	179	325	825	0	923	0	19	2320	5.3	2.4
	173	385	698	892	233	0	19	2320	5.3	3.5
	173	385	742	515	515	0	19	2320	5.3	2.9
	173	385	746	0	963	0	19	2320	5.3	2.5

References

[1] J. M. V. Gómez-Soberón, "Porosity of recycled concrete with substitution of recycled concrete aggregate: An experimental study," *Cement and Concrete Research,* vol. 32, no. 8, pp. 1301-1311, 2002/08/01/ 2002, doi: https://doi.org/10.1016/S0008-8846(02)00795-0.

[2] M. Etxeberria, A. R. Marí, and E. Vázquez, "Recycled aggregate concrete as structural material," *Materials and Structures,* journal article vol. 40, no. 5, pp. 529-541, June 01 2007, doi: 10.1617/s11527-006-9161-5.

[3] L. Evangelista and J. de Brito, "Mechanical behaviour of concrete made with fine recycled concrete aggregates," *Cement and Concrete Composites,* vol. 29, no. 5, pp. 397-401, 2007/05/01/ 2007, doi: https://doi.org/10.1016/j.cemconcomp.2006.12.004.

[4] S. C. Kou, C. S. Poon, and D. Chan, "Influence of Fly Ash as Cement Replacement on the Properties of Recycled Aggregate Concrete," *Journal of Materials in Civil Engineering,* vol. 19, no. 9, pp. 709-717, 2007, doi: doi:10.1061/(ASCE)0899-1561(2007)19:9(709).

[5] S. C. Kou, C. S. Poon, and D. Chan, "Influence of fly ash as a cement addition on the hardened properties of recycled aggregate concrete," *Materials and Structures,* vol. 41, no. 7, pp. 1191-1201, 2008.
[6] K.-H. Yang, H.-S. Chung, and A. F. Ashour, "Influence of Type and Replacement Level of Recycled Aggregates on Concrete Properties," 2008.
[7] R. Kumutha and K. Vijai, "Strength of concrete incorporating aggregates recycled from demolition waste," *ARPN Journal of Engineering and Applied Sciences,* vol. 5, no. 5, pp. 64-71, 2010.
[8] C. J. Zega and A. A. D. Maio, "Recycled Concretes Made with Waste Ready-Mix Concrete as Coarse Aggregate," *Journal of Materials in Civil Engineering,* vol. 23, no. 3, pp. 281-286, 2011, doi: doi:10.1061/(ASCE)MT.1943-5533.0000165.
[9] G. Fathifazl, A. G. Razaqpur, O. B. Isgor, A. Abbas, B. Fournier, and S. Foo, "Creep and drying shrinkage characteristics of concrete produced with coarse recycled concrete aggregate," *Cement and Concrete Composites,* vol. 33, no. 10, pp. 1026-1037, 2011.
[10] M. C. Rao, S. Bhattacharyya, and S. Barai, "Influence of field recycled coarse aggregate on properties of concrete," *Materials and Structures,* vol. 44, no. 1, pp. 205-220, 2011.
[11] A. Abd Elhakam, A. E. Mohamed, and E. Awad, "Influence of self-healing, mixing method and adding silica fume on mechanical properties of recycled aggregates concrete," *Construction and Building Materials,* vol. 35, pp. 421-427, 2012/10/01/ 2012, doi: https://doi.org/10.1016/j.conbuildmat.2012.04.013.
[12] P. Pereira, L. Evangelista, and J. De Brito, "The effect of superplasticizers on the mechanical performance of concrete made with fine recycled concrete aggregates," *Cement and concrete composites,* vol. 34, no. 9, pp. 1044-1052, 2012.
[13] L. Butler, J. S. West, and S. L. Tighe, "Effect of recycled concrete coarse aggregate from multiple sources on the hardened properties of concrete with equivalent compressive strength," *Construction and Building Materials,* vol. 47, pp. 1292-1301, 2013.
[14] C. Thomas, J. Setién, J. A. Polanco, P. Alaejos, and M. Sánchez de Juan, "Durability of recycled aggregate concrete," *Construction and Building Materials,* vol. 40, pp. 1054-1065, 2013/03/01/ 2013, doi: https://doi.org/10.1016/j.conbuildmat.2012.11.106.
[15] K. H. Younis and K. Pilakoutas, "Strength prediction model and methods for improving recycled aggregate concrete," *Construction and Building Materials,* vol. 49, pp. 688-701, 2013/12/01/ 2013, doi: https://doi.org/10.1016/j.conbuildmat.2013.09.003.
[16] G. Andreu and E. Miren, "Experimental analysis of properties of high performance recycled aggregate concrete," *Construction and Building Materials,* vol. 52, pp. 227-235, 2014/02/15/ 2014, doi: https://doi.org/10.1016/j.conbuildmat.2013.11.054.
[17] Z. H. Duan and C. S. Poon, "Properties of recycled aggregate concrete made with recycled aggregates with different amounts of old adhered mortars," *Materials & Design,* vol. 58, pp. 19-29, 2014.
[18] P. Folino and H. Xargay, "Recycled aggregate concrete – Mechanical behavior under uniaxial and triaxial compression," *Construction and Building Materials,* vol. 56, pp. 21-31, 2014/04/15/ 2014, doi: https://doi.org/10.1016/j.conbuildmat.2014.01.073.
[19] D. Pedro, J. de Brito, and L. Evangelista, "Performance of concrete made with aggregates recycled from precasting industry waste: influence of the crushing process," *Materials and Structures,* journal article vol. 48, no. 12, pp. 3965-3978, December 01 2015, doi: 10.1617/s11527-014-0456-7.
[20] C. Thomas, I. Sosa, J. Setién, J. A. Polanco, and A. I. Cimentada, "Evaluation of the fatigue behavior of recycled aggregate concrete," *Journal of Cleaner Production,* vol. 65, pp. 397-405, 2014/02/15/ 2014, doi: https://doi.org/10.1016/j.jclepro.2013.09.036.

6. Randomization tests

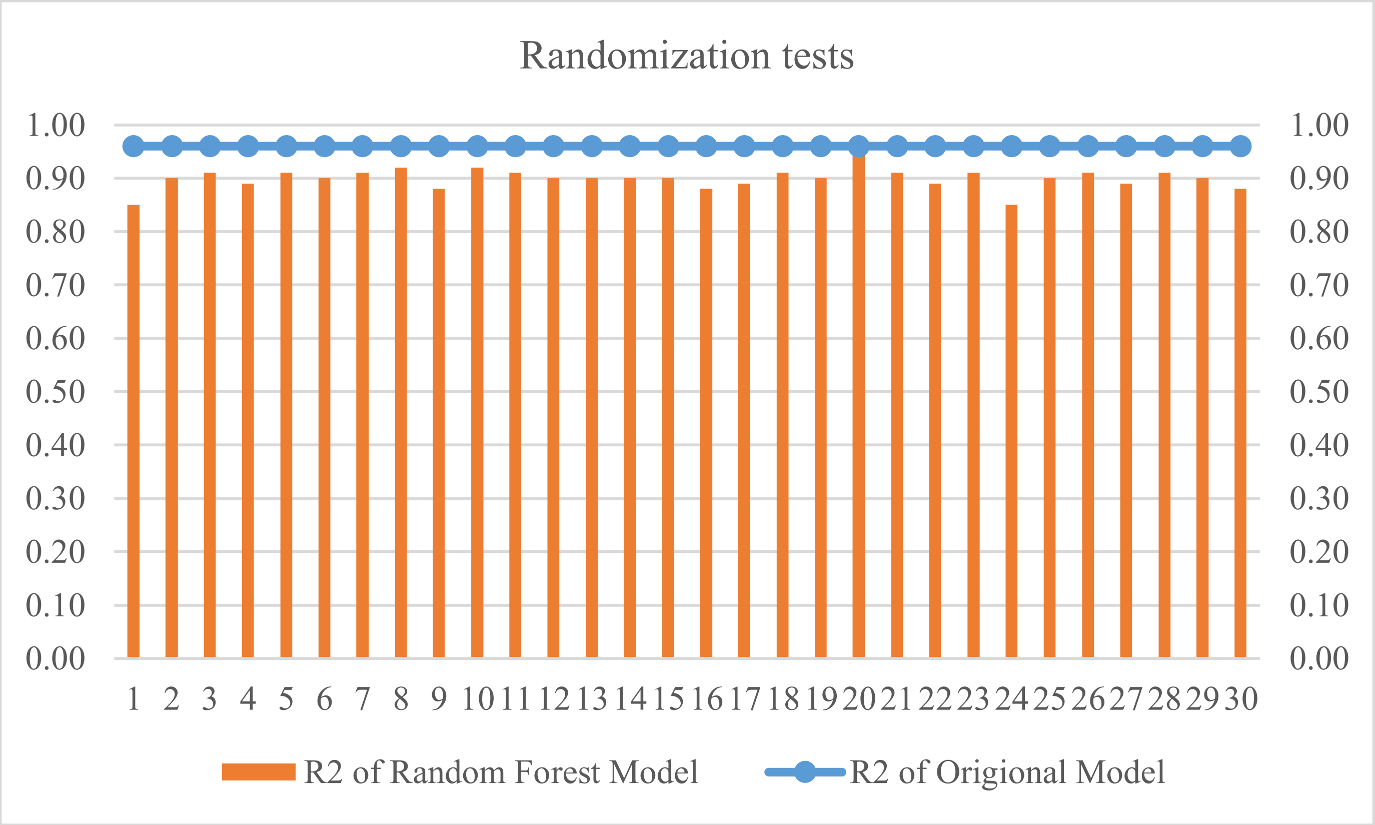

Figure S1. RF randomization test.
